# A shortcut for multiple testing on the directed acyclic graph of gene ontology

**DOI:** 10.1186/s12859-014-0349-3

**Published:** 2014-11-01

**Authors:** Garrett Saunders, John R Stevens, S Clay Isom

**Affiliations:** Utah State University, Department of Mathematics & Statistics, Logan, Utah USA; Utah State University, Department of Animal, Dairy, and Veterinary Sciences, Logan, Utah USA; Brigham Young University-Idaho, Department of Mathematics, Rexburg, Idaho USA

**Keywords:** Bonferroni, Holm, Gene ontology, Multiple testing

## Abstract

**Background:**

Gene set testing has become an important analysis technique in high throughput microarray and next generation sequencing studies for uncovering patterns of differential expression of various biological processes. Often, the large number of gene sets that are tested simultaneously require some sort of multiplicity correction to account for the multiplicity effect. This work provides a substantial computational improvement to an existing familywise error rate controlling multiplicity approach (the Focus Level method) for gene set testing in high throughput microarray and next generation sequencing studies using Gene Ontology graphs, which we call the Short Focus Level.

**Results:**

The Short Focus Level procedure, which performs a shortcut of the full Focus Level procedure, is achieved by extending the reach of graphical weighted Bonferroni testing to closed testing situations where restricted hypotheses are present, such as in the Gene Ontology graphs. The Short Focus Level multiplicity adjustment can perform the full top-down approach of the original Focus Level procedure, overcoming a significant disadvantage of the otherwise powerful Focus Level multiplicity adjustment. The computational and power differences of the Short Focus Level procedure as compared to the original Focus Level procedure are demonstrated both through simulation and using real data.

**Conclusions:**

The Short Focus Level procedure shows a significant increase in computation speed over the original Focus Level procedure (as much as ∼15,000 times faster). The Short Focus Level should be used in place of the Focus Level procedure whenever the logical assumptions of the Gene Ontology graph structure are appropriate for the study objectives and when either no *a priori* focus level of interest can be specified or the focus level is selected at a higher level of the graph, where the Focus Level procedure is computationally intractable.

**Electronic supplementary material:**

The online version of this article (doi:10.1186/s12859-014-0349-3) contains supplementary material, which is available to authorized users.

## Background

Microarray technology and next generation sequencing have played an important role in discovering important associations between gene expression patterns and phenotype [1]. Such gene expression technologies have been instrumental in discoveries ranging from the retarding of aging in mice brought about by caloric restrictions in diet [2] to the identification of various types of diffuse large B-cell lymphoma in humans [3]; from characterizing the transcriptomes of *in vitro* maturated porcine embryos [4] to uncovering the underlying genes and pathways involved in Alzheimer’s disease [5]. While both microarray and next generation sequencing technologies allow researchers to study the differential expression of genes across conditions or treatments, each has their advantages and disadvantages [1]. However, in either case, the resulting increase in genetic knowledge has allowed researchers to group genes with common function into gene sets and test these gene sets for differential expression [6,7].

One rich source of gene set knowledge is found in the Gene Ontology database [8]. The Gene Ontology (GO) provides a controlled vocabulary that is not specific to any particular species. This vocabulary is divided into three general ontologies, Molecular Function (MF), Cellular Component (CC), and Biological Process (BP). Individual GO Terms form the basis of these vocabularies and are structured through parent child relationships with more general terms as parents and more specific terms as children. Each GO Term typically contains a definition of its biological process (molecular function or cellular component) and other annotation as well as a mapping of all known gene products involved in its specified process (function or component). For convenience in presentation, the remainder of this work will focus on the biological process ontology.

Gene set testing allows for the quantification of the significance of activity level differences between treatment groups for specific biological processes of interest. For example, a recent study on human longevity compared the gene expression profiles corresponding to 1,808 different biological processes for nonagenarians and a control group to identify 73 biological processes associated with longevity [9]. When there are relatively few gene sets (biological processes) of *a priori* interest (1,808 in [9]), the impact of the multiplicity correction for the tests of differential expression (or differential activity) of the gene sets can be greatly lessened as compared to individually testing all member genes (45,164 in [9]), improving the power of the test. Even when no *a priori* gene set of interest can be specified, it can still be highly beneficial to test all known gene sets from a biological process database for differential expression, as the number of gene sets is still typically magnitudes smaller than the corresponding number of individual genes [6,10].

Many methods of gene set testing have been proposed in the literature as reviewed in [11]. These can essentially be divided into two classes of gene set testing, often referred to as *competitive* tests and *self contained* tests. The *competitive* tests compare the expression profiles of the genes in the set to those not in the set. The *self contained* tests focus only on those genes within the set and compares them to some fixed standard. While the first are more popular [7,12], the second have been shown to be more powerful [11,13]. Further, the null hypothesis associated with the *self contained* tests,

$H_{0}^{self}$*: no genes in the gene set are differentially expressed,*

has been shown to be the more logical generalization of single gene testing (with other advantages that will be explained later on) as compared to the *competitive* test null hypothesis

$H_{0}^{comp}$*: the genes in the gene set are at most as often differentially expressed as the genes in the complement of the gene set.*

While gene set testing methods are varied in their approach, they are alike in that they test each GO term, i.e. gene set, individually. Thus, when more than one GO term is tested simultaneously (typically hundreds or thousands are tested simultaneously) some sort of multiplicity adjustment is necessary to preserve control over either the familywise error rate (FWER) or the false discovery rate (FDR) or a derivative of these error rates. The FDR is typically the error rate of choice in exploratory studies where follow up confirmatory studies are then conducted [14]. On the other hand, the FWER is typically the suggested error rate for confirmatory studies [15]. We also suggest that the FWER is highly appropriate for exploratory gene set studies as, in our experience, it is seldom *more* results that are desired, but the *most promising* real significances that are sought. The FWER offers the best error rate control for such conclusions [15].

The *Focus Level* method is a powerful method of multiplicity adjustment for *self contained* gene set testing, which takes into account the structure of the GO graph while controlling (strongly) the FWER [10]. This approach is more powerful than standard FWER controlling methods such as the Bonferroni and uniformly more powerful Bonferroni-Holm [16] procedures for multiple testing with GO graphs [10]. However, it is important to note that this increase in power comes at the cost of requiring the logical structure of the GO graph to become part of the multiplicity adjustment.

The Focus Level method allows the researcher to select the level of the GO graph in which they are most interested. This is called the *focus level*. The procedure then applies a *top-down* and *bottom-up* approach from the specified focus level. First, the terms in the focus level are tested using the Bonferroni-Holm adjustment [16]. Then, in the bottom-up approach, any term above the focus level is declared significant when any of its offspring in the focus level have been declared significant. This inheritance of *P*-values is accomplished through the assumption that a parent term must be differentially expressed if any of its children terms are differentially expressed, a logical assumption for the GO graph structure [10]. In the top-down procedure, significance of the children of the focus level terms is decided through an application of the closed testing procedure of [17].

While the Focus Level method is a powerful approach to adjusting for multiplicity, it quickly becomes computationally infeasible when the selected focus level contains a large number of offspring in the GO graph [10]. This computational limitation makes it essentially impossible to perform the full top-down approach, a rather significant disadvantage [18]. Using the full top-down approach provides researchers the default focus level of the root node (GO:0008150 in the context of the BP GO ontology) whenever they have no *a priori* interest in a given focus level, a common scenario, see for example [18]. This also allows adjusted *P*-values to be considered apart from their context in the GO graph which is advantageous to reporting on single significant gene sets of interest. Discussions of the significant findings of the Focus Level method are currently restricted to their context within the GO graph [10].

This work proposes an improvement to the top-down portion of the Focus Level method [10] which we call the Short Focus Level as it performs a shortcut of the full Focus Level method. This is accomplished using a novel application of the general graphical Bonferroni adjustment for multiple testing as proposed by [19], which is a generalization of closed testing based on weighted Bonferroni tests [20]. The Short Focus Level procedure shows a significant improvement in computational speed (as much as ∼ 15,000 times faster) while maintaining similar power to that of the original Focus Level procedure and even showing a gain in power over the original Focus Level procedure for certain scenarios while experiencing a loss in power for others. Most importantly, the computational improvements are such that the full top-down method can now be performed on a standard operating system within just a few minutes. The R code [21] for the Short Focus Level procedure is included in the mvGST package [22,23]; see also Additional files 1 and 2.

## Methods

The Focus Level procedure [10] adjusts for multiple gene set tests using the structure of the directed acyclic graphs of the Gene Ontology (GO). Two basic assumptions underly the method.

1. A non-differentially expressed parent gene set implies the children gene sets are also non-differentially expressed.
2. If the children gene sets form a partition of the parent gene set and are all non-differentially expressed, then the parent gene set is also non-differentially expressed.

These assumptions ensure coherence of the resulting significant subgraph and facilitate interpretations [10]. Note that these assumptions are necessary if the objective is to control the FWER within the structure of the GO graph. If preserving the graph structure in the multiplicity correction is not of interest to the researcher, then the FWER (or even the false discovery rate) could be controlled by the standard Holm’s correction [16] (or any false discovery rate controlling method which allows for arbitrary dependence structures), however such an approach will result in a loss of coherence for the significant subgraph.

Assumptions A1 and A2 require that the null hypothesis for each gene set is that no genes in the gene set are differentially expressed. The alternative in each case being that at least one gene in the set is differentially expressed. Thus, only self contained gene set testing methods (which utilize this hypothesis framework) can be used to test the gene sets of the GO graph if the Focus Level method of multiplicity adjustment is used. This excludes gene set enrichment methods such as those proposed in [12] but supports very well the Global Test of [6], *P*-value combination methods such as Fisher’s and Stouffer’s methods [13,24], as well as Global Ancova [25], PLAGE [26], and SAM-GS [27].

As prescribed by [10] there are two requirements in the selection of the focus level. These requirements are labeled “FL1” and “FL2” here for later reference in subsection “The *Short* focus level procedure”.

1. No offspring of a focus level term be contained in the focus level.
2. All remaining terms are either ancestors or offspring of the focus level terms.

**Figure 1:**
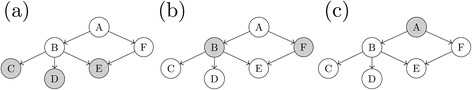
**Three possible focus levels (filled nodes) for a simplified example toy GO graph.** **(a)** Full bottom-up approach. **(b)** Intermediate focus level. **(c)** Full top-down approach.

Figure 1 demonstrates on a simplified toy GO graph how the focus level (filled nodes) could be chosen. The full bottom-up approach (panel (a) of Figure 1) selects all GO Terms corresponding to terminal nodes as the focus level, in this example, nodes *C*, *D*, and *E*. The full top-down approach (panel (c) of Figure 1) selects the root node, *A* in this case, as the focus level. Finally, in a typical GO graph there are many (hundreds or thousands) of options for focus levels contained somewhere in the middle of the GO graph. In the simplified example graphs of Figure 1 the most logical intermediate focus level is demonstrated with nodes *B* and *F* (panel (b)). It would also be possible to use nodes *C*, *D* and *F* as the focus level but such choices in actual GO graphs do not provide a consistent *level* of specificity in the graph and would not be as logical a choice. Choosing nodes *C*, *D*, *E*, and *F* as the focus level would not be allowed as *E* is a child of *F*, violating the requirement that the focus level must not contain any offspring of another focus level term (*E* is an offspring term of *F*). Choosing only node *B* as the focus level would also not be allowed as node *F* is neither an ancestor or offspring term of *B*, violating the second requirement.

The top-down portion of the Focus Level procedure of [10], which applies the closed testing approach of [17], requires closing the GO graph under all unions from the focus level down. This is done by treating each focus level term, along with all of its offspring terms, as separate graphs which are each closed under all possible unions. As these separate closed graphs will share common elements, the full closed graph $\tilde {G}$ is obtained by unioning each of the separately closed graphs into a single graph which is also unioned to all ancestor terms of the focus level.

**Figure 2:**
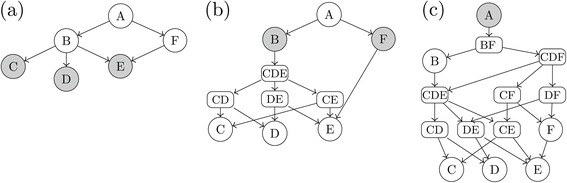
**Closures of the GO graphs from Figure** 1 **where the filled nodes represent the different choices of the focus level.** **(a)** Full bottom-up approach. **(b)** Intermediate focus level. **(c)** Full top-down approach.

To demonstrate, consider the closures of each of the example GO graphs from Figure 1 as shown in Figure 2. In each case, the nodes above the focus level remain unchanged, while the creation of several sets not present in the original example GO graph (depicted with rounded rectangles) are required in order to close the graph under all possible unions from the focus level down. Since the closing of the graph is only required from the selected focus level down, it is clear from Figure 2 that the more offspring terms the focus level contains, the greater the number of sets that must be created to close the graph. Closing the graph can quickly become computationally infeasible in practice. Importantly, performing the full top-down approach as in panel (c) of Figure 2 is rarely possible in real applications due to the computational burden.

To partially amend the computational difficulties of the Focus Level method, [10] implement a more efficient method of computing the closed graph using what they term “atom sets”. These atom sets form a core collection of gene sets which form a basis for all gene sets in the graph. All other gene sets in the graph (as well as its closure) can be created through unions of the atom sets. This ensures the size of the closed graph is 2^*k*^−1, where *k* is the number of atom sets, which is often smaller (and never larger) than the size of the original closed graph. Further, [10] recommend selecting the focus level so that no more than 9-12 atom sets are required to recreate the offspring of any single focus level term. They also suggest computing only the smallest few adjusted *P*-values to save computation time in place of computing all adjusted *P*-values.

This work offers an alternative solution to improve on the computational speed of the top-down portion of the Focus Level method through an application of the general graphical Bonferroni adjustment of [19]. This allows for a short-cut of length *m* in place of the currently applied full closed testing approach of [17]. In the following section, we summarize the general graphical Bonferroni adjustment approach and show how we tailor the method for a powerful application to the top-down portion of the Focus Level method.

### The graphical Bonferroni adjustment

A powerful and versatile graphical generalization of weighted Bonferroni based closed testing [17] which provides strong control of the familywise error rate (FWER) at a specified level *α* was proposed in [19]. Their approach represents all *m* hypotheses of interest, *H*_1_,…,*H*_*m*_ as nodes in a directed graph. Each node can be thought of here as a gene set, with a corresponding hypothesis *H*_*i*_ testing for differential expression. Node *i*, representing hypothesis *H*_*i*_, is allocated a local threshold *α*_*i*_ for all *i*=1,…,*m*. Nodes are joined by edges with weights *g*_*ij*_ dictating the proportion of the local threshold *α*_*i*_ that is allocated to all connected hypotheses (nodes) *H*_*j*_ in the case that hypothesis *H*_*i*_ is rejected. The structure of the graph as well as the size of the local thresholds *α*_*i*_ and edge weights *g*_*ij*_ is dependent on the objectives of the study. The versatility of the method is in the generality of the regularity conditions and updating algorithm for the directed graph. The regularity conditions require the following [19]:

1. The local thresholds *α*_1_,…,*α*_*m*_ satisfy $\sum _{i=1}^{m} \alpha _{i} \leq \alpha $.
2. The edge weights satisfy 0≤*g*_*ij*_≤1, *g*_*ii*_=0, and $\sum _{k=1}^{m} g_{\textit {ik}} \leq 1$ for all *i*,*j*=1,…,*m*.

The updating algorithm defines a sequentially rejective test procedure and is given as follows [19]. Note that *p*_*i*_ represents the observed *P*-value for the test of hypothesis *H*_*i*_.



The proof that Algorithm 1 defines a sequentially rejective closed testing procedure which strongly controls the FWER at level *α* is found in the Appendix of [19], and depends directly on Theorem 1 from [20]. Both [28] and [29] claim that Theorem 1 from [20] cannot be directly applied to the hypotheses of the GO graph as the hypotheses are nested, creating logical restrictions. In their own words, [28] claim that “the shortcut procedure of [20] cannot be applied to restricted hypotheses”. Similarly, [29] state, “these methods [19] cannot make use of logical relationships between hypotheses and, as such, do not incorporate graph-based methods which exploit such relationships, such as [the Focus Level procedure] of [10]”. However, in the following we present a restricted hypotheses example where the methods of [19] can be applied. The following section sets forward some important notation and vocabulary and then demonstrates that while these claims are technically true, the methods of [19] can be applied to the Focus Level method if one of the assumptions underlying Theorem 1 of [20] is slightly relaxed. We prove this with Theorem 1.

### Restricted hypotheses example

Let *H*_1_,…,*H*_*m*_ denote *m* hypotheses of interest and call these the elementary hypotheses. Let *I* denote a non-empty index set such that *I*⊆{1,…,*m*} and denote an intersection hypothesis by *H*_*I*_ where *H*_*I*_=∩_*i*∈*I*_*H*_*i*_. The closed test procedure [17] utilizes the intersection closed set of hypotheses $\mathcal {H}=\{H_{I}: I\subseteq \{1,\ldots,m\}, I\neq \emptyset \}$. In the case that the hypotheses are unrestricted, $|\mathcal {H}|=2^{m}-1$ and Algorithm 1 of [19] is proven to hold. On the other hand, the hypotheses are restricted if for index sets *I* and *J* it is true that *I*≠*J* and *H*_*I*_=*H*_*J*_ so that $|\mathcal {H}|< 2^{m}-1$. In this case, Algorithm 1 cannot currently be applied [28,29].

As the hypotheses corresponding to any GO graph are always restricted, the methods of [19] cannot be applied to the GO graph under the current framework. However, the following closed test example from [28] can be extended to demonstrate how Algorithm 1 *can* be applied to the case of restricted hypotheses. This example sets the stage for Theorem 1, where we relax the assumptions of [20] to formally establish how the methods of [19] can indeed be applied to restricted hypotheses, and hence, the GO graph.

Consider the partially nested elementary hypotheses *H*_1_, *H*_2_, *H*_3_, and *H*_4_ defined as follows for the parameters *θ*_1_ and *θ*_2_ where *δ*_1_,*δ*_2_>0.

(1) $$ {}H_{1}\!:\! \theta_{1} \leq -\delta_{1}, \quad H_{2}\!:\! \theta_{1} \leq 0, \quad H_{3}\!:\! \theta_{2} \leq -\delta_{2}, \quad H_{4}\!:\! \theta_{2} \leq 0  $$

The full closure family of hypotheses  of these four elementary hypotheses would contain 2^4^−1=15 distinct intersection hypotheses if they were unrestricted. However, the restrictions stemming from the partial nesting of *H*_1_ with *H*_2_ (*H*_1_⊂*H*_2_) and *H*_3_ with *H*_4_ (*H*_3_⊂*H*_4_) reduce the final closure to just eight distinct intersection hypotheses. For example, *H*_12_=*H*_1_∩*H*_2_=*H*_1_ and *H*_34_=*H*_3_∩*H*_4_=*H*_3_. Computing all intersections and retaining only the disctinct intersection hypotheses shows

(2) $$ \mathcal{H} = \left\{ H_{1}, H_{2}, H_{3}, H_{4}, H_{13}, H_{14}, H_{23}, H_{24} \right\}.  $$

**Figure 3:**
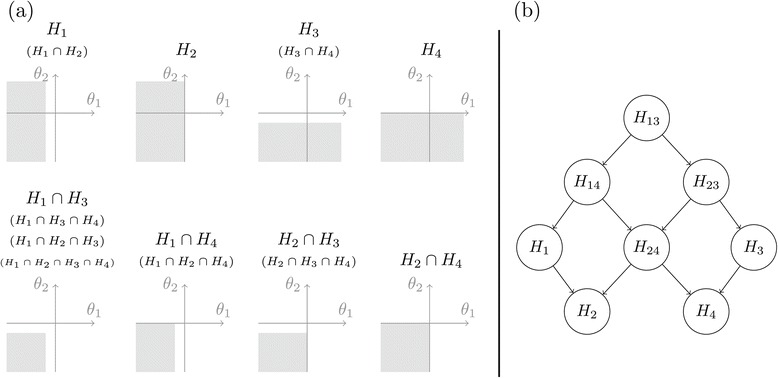
**Visualization of elementary hypotheses and their closure.** **(a)** Graphical demonstration of the elementary hypotheses ***H***_***1***_ ***,…,H***_***4***_ and distinct intersection hypotheses. The null parameter space is shaded in gray for each hypothesis. Redundant intersection hypotheses are written in parentheses. **(b)** The closed test approach given the structure of the hypotheses. Testing begins with *H*_13_, the full intersection hypothesis, and terminates at or before testing *H*_2_ and *H*_4_.

Each of the null parameter spaces corresponding to the hypotheses in  are graphically depicted in panel (a) of Figure 3.

A closed test approach to  is given in [28] which begins with the raw *p*-values *p*_1_,*p*_2_,*p*_3_, and *p*_4_ obtained from testing the original elementary hypotheses *H*_1_,*H*_2_,*H*_3_, and *H*_4_, each with *α*-level tests, respectively. To define the closed test approach, they compute the closed test *p*-values $p_{H_{i}}$ for each hypotheses *H*_*i*_ in  by the following rules. First, $p_{H_{1}} = p_{1}$ and $p_{H_{3}} = p_{3}$. Second, $p_{H_{2}} = \max \{p_{1},p_{2}\}$ and $p_{H_{4}} = \max \{p_{3},p_{4}\}$. Finally, $p_{H_{\textit {ij}}} = \min \{1,2p_{H_{i}},2p_{H_{j}}\}$, *i*=1,2 and *j*=3,4. The closed test procedure [17] is then applied to  as depicted in panel (b) of Figure 3 using the closed test *p*-values $p_{H_{i}}$ as explained in the following paragraph.

**Figure 4:**
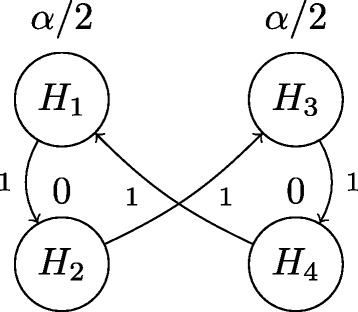
**Graphical Bonferroni adjustment approach for the partially nested elementary hypotheses** ***H***_***1***_ ***,…,H***_***4***_ **which performs the closed test described in [** 28 **] when Algorithm 1 is applied to the graph.**

The closed test procedure only tests a hypothesis $H_{i}\in \mathcal {H}$ if all hypotheses implying *H*_*i*_ are first rejected. For example, *H*_1_ can only be tested by the closed test procedure if *H*_13_ and *H*_14_ are first rejected, see panel (b) of Figure 3. In other words, the hypothesis corresponding to a child node is only tested if its parent node hypothesis is first rejected [28] state that, “this closed test procedure controls the familywise error rate strongly at level *α* and reflects the logical constraints among the elementary hypotheses”. We show that this closed test approach for these restricted hypotheses can be performed using the directed graph of Figure 4 and Algorithm 1 from [19].

**Figure 5:**
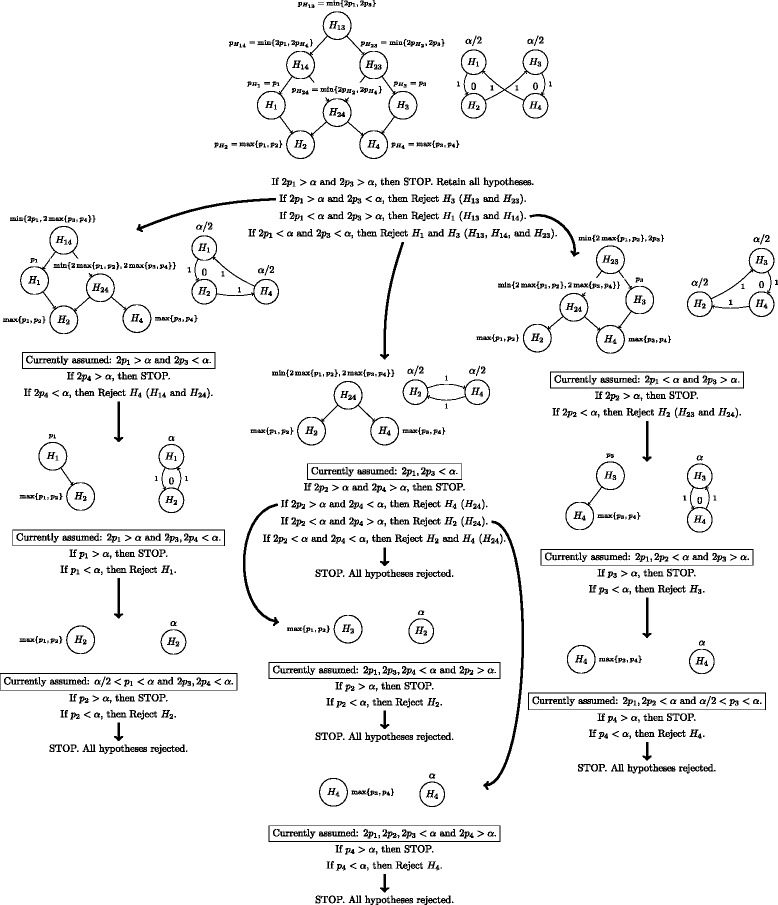
**Flow chart demonstration of the equivalence of the graphical shortcut tailored from the methods of [** 19 **] to that of the full closed test procedure proposed in [** 28 **] within the context of the previously established restricted hypotheses example.** At each step in the chart, the left graph represents the full closed test approach, while the right graph depicts the graphical shortcut.

Consider the sequential rejection procedure resulting from the application of Algorithm 1 [19] to the directed graph shown in Figure 4. Initial local thresholds of *α*/2 are assigned to *H*_1_ and *H*_3_ and local thresholds of zero assigned to *H*_2_ and *H*_4_ as depicted in Figure 4. The weighted edges provide for the reallocation of the local thresholds in the case of rejection of either *H*_1_ or *H*_3_. If neither *H*_1_ nor *H*_3_ can be rejected at the *α*/2-level, then the testing is stopped with no rejections. This corresponds to the first step of the closed test procedure described previously, as proposed in [28]. As can be seen in panel (b) of Figure 3, the closed test requires the rejection of the intersection hypothesis *H*_13_ before any other rejection can occur. This requires that the previously defined closed test *p*-value $p_{H_{13}} = \min \{2p_{H_{1}},2p_{H_{3}}\}$ satisfy $p_{H_{13}}<\alpha $. Since $p_{H_{1}}$ and $p_{H_{3}}$ were defined to be *p*_1_ and *p*_3_ respectively for this particular example, it follows that $p_{H_{13}}<\alpha $ implies 2 min{*p*_1_,*p*_3_}<*α*, witnessing that the methods agree on their starting analysis using only the values of *p*_1_ and *p*_3_. The flow chart in Figure 5 further demonstrates that the two approaches agree for all possible test scenarios and hence, that the shortcut of [19] can successfully be applied to this example of restricted hypotheses.

### Definitions and preliminaries to Theorem 1

A deeper inspection of Figure 5 will reveal the reason why the shortcut from [19] can be applied to the example of restricted hypotheses of the previous section. To explain how, we must first define two terms, *consonance* and *natural consonance*.

The traditional definition of *consonance* [30] relies on the idea of maximal hypotheses. It states that consonance is the property of certain closed tests where rejection of an intersection hypothesis $H_{i}\in \mathcal {H}$ implies rejection of a maximal hypothesis $H\in \mathcal {H}$. Here, a maximal hypothesis $H\in \mathcal {H}$ is such that there is no $H'\in \mathcal {H}$ with *H*^′^⊃*H*. (When the closed test corresponding to the hypotheses in  is depicted graphically, as in panel (b) of Figure 3, in can be seen that maximal hypotheses correspond to the leaf nodes of the graph. Further, in context of the GO graph, maximal hypotheses correspond to the leaf nodes of the graph, while the minimal hypothesis corresponds to the root node of the graph). From the example of the previous section, it can be seen that only *H*_2_ and *H*_4_ are maximal. Thus, the closed test of the example is not consonant in the traditional sense as rejection of the intersection hypothesis *H*_13_ does not imply the rejection of either of the maximal hypotheses *H*_2_ or *H*_4_.

*Natural consonance* is a similar, but slightly more relaxed property than *consonance*, and differs in that it implies the rejection of only an elementary hypothesis (not necessarily a leaf node in the closure graph) whenever any other hypothesis $H_{i}\in \mathcal {H}$ is first rejected. This relaxed definition is more recent and is due to [28]. Importantly, it is easier for a closed test to satisfy the property of *natural consonance* than that of *consonance*. The claims of both [28] and [29] that Algorithm 1 [19] is not applicable to restricted hypotheses rest on the subtle difficulty of how consonance is defined. Note (v) following Theorem 2 of [28] claims that “consonance with respect to the elementary hypotheses [natural consonance] always implies the existence of a nested shortcut of size *m*”, where *m* is the number of elementary hypotheses. The *natural consonance* of the closed test allows for the shortcut from [19] to be applied to the restricted hypothesis example of the previous section, as explained in the following paragraph.

Examining the flow chart of Figure 5 will reveal that the closed test procedure proposed by [28] has this property of consonance with respect to the elementary hypotheses *H*_1_, *H*_2_, *H*_3_, and *H*_4_, i.e., the closed test for this example is *naturally consonant*. This follows from the fact that rejection of the intersection hypothesis *H*_13_ implies rejection of either of the hypotheses *H*_1_ or *H*_3_ which are two of the original four elementary hypotheses. Note as before that rejection of *H*_13_ requires that either 2*p*_1_<*α* or 2*p*_3_<*α* by the definition of $p_{H_{13}}$. If say 2*p*_1_<*α*, then *H*_13_ is rejected. Further, since 2*p*_1_<*α*, *H*_14_ is also rejected as $p_{H_{14}} = \min \{1,2p_{H_{1}},2p_{H_{4}}\}=\min \{2p_{1},2p_{H_{4}}\}<\alpha $. Most importantly, 2*p*_1_<*α* provides for *H*_1_ to be rejected, as the closed test *p*-value $p_{H_{1}}$ requires only *p*_1_<*α* which is certainly satisfied if 2*p*_1_<*α*. Hence, in this case, the rejection of the intersection hypothesis *H*_13_ implied rejection of the elementary hypothesis *H*_1_. A similar scenario holds for the elementary hypothesis *H*_3_ if 2*p*_3_<*α* instead of (or as well as) 2*p*_1_<*α*. Finally, rejection of *H*_24_ similarly implies rejection of either *H*_2_ or *H*_4_. Thus, the closed test procedure for these restricted hypotheses admits the shortcut of [19] because of the consonance of the closed test with respect to the elementary hypotheses, i.e. the closed test is naturally consonant.

We now extend Theorem 1 of [20] to restricted hypotheses, and thereby verify the appropriateness of the graphical shortcut of [19] for restricted hypotheses. To this end, let *m* elementary hypotheses *H*_1_,…,*H*_*m*_ of interest be given and denote by  their closure under intersection. For the purposes of Theorem 1,  can be either restricted or unrestricted. Let *α*_*i*_(*I*) denote the local significance levels for an intersection hypothesis $H_{i}\in \mathcal {H}$ where $\sum _{i\in I} \alpha _{i} \leq \alpha $ for all non-empty *I*⊆{1,…,*m*}.

#### **Theorem1**.

(Extension of Theorem 1 from [20] to restricted hypotheses.) If for *∅*≠*I*,*J*⊆{1,…,*m*} with *∅*≠*H*_*I*_⊂*H*_*J*_ it holds that *α*_*i*_(*I*)≤*α*_*i*_(*J*), then the closed test for  based on local Bonferroni tests is naturally consonant and a shortcut equivalent to the following procedure is possible (adapted from [19]).

0. Set *M*={1,…,*m*}.
1. Set *I* equal to the smallest subset of *M* such that *H*_*I*_=*H*_*M*_.
2. Reject *H*_*j*_ if there exists *j*∈*I* such that *p*_*j*_≤*α*_*j*_(*I*). If no such *j* exists, then stop.
3. Set *M*→*M*∖*j*.
4. If |*M*|≥1 return to Step 1. Otherwise, stop.

#### *Proof*.

First, note that in the case of unrestricted hypotheses, natural consonance and consonance are identical [28] so that the proof is already demonstrated in Theorem 1 of [20]. Consider then the case of restricted hypotheses in the sense that for *∅*≠*I*,*J*⊆{1,…,*m*} with *I*≠*J* it is true that *∅*≠*H*_*I*_=*H*_*J*_ so that $|\mathcal {H}|<2^{m}-1$. Then, for *I*,*J* with *∅*≠*H*_*I*_⊂*H*_*J*_ it follows from *α*_*i*_(*I*)≤*α*_*i*_(*J*) that *p*_*j*_≤*α*_*j*_(*I*) implies *p*_*j*_≤*α*_*j*_(*J*). Thus, rejection of *H*_*I*_ implies rejection of some elementary hypothesis *H*_*j*_, witnessing that the closed test for  is indeed naturally consonant.

### Discussion of Theorem 1

Some comments are in order regarding Theorem 1. First, while an intersection hypothesis *H*_*I*_ may not be unique in , it must not be empty for the nested shortcut of length *m* to exist. Second, the only difference between the proof here and the proof for unrestricted hypotheses [20] is in the definition of consonance. Here we follow the suggestion in [28] and allow natural consonance, which can be seen as a loosening of the requirements of consonance to include all elementary hypotheses instead of just all maximal hypotheses. The important distinction is that for unrestricted hypotheses, all elementary hypotheses are maximal. The same is not necessarily true for restricted hypotheses. Third, as in the previous restricted hypotheses example, restricted hypotheses are often the result of nested elementary hypotheses. This is certainly the case for the hypotheses attached to the gene sets of the GO graphs. Fourth, the main importance of the extended Theorem 1 rests with its assurance that a naturally consonant closed test based on weighted Bonferroni tests exists so long as the monotonicity condition *α*_*i*_(*I*)≤*α*_*i*_(*J*) is satisfied for all *∅*≠*H*_*i*_⊂*H*_*J*_ in . Fifth, Theorem 1 does not specify that *any* graph with local thresholds of *α*=(*α*_1_,…,*α*_*m*_) and edge weights **G**={*g*}_*ij*_, denoted by (*α*,**G**), can combine with Algorithm 1 and lead to a consonant closed test. It simply specifies the conditions under which a consonant closed test based on local Bonferroni tests can be formed.

One important rule on the graph (*α*,**G**) when the hypotheses are restricted is that the local threshold *α*_*i*_ for an elementary hypothesis *H*_*i*_ must remain zero until all elementary hypotheses *H*_*j*_ with *H*_*j*_⊂*H*_*i*_ are first rejected. This property can be seen to hold for the graph of Figure 4. However, if the graph in Figure 4 allowed for any of *H*_1_’s threshold to be passed to *H*_4_ or similarly, if *H*_3_ allowed for anything to be passed to *H*_2_, this property would no longer hold. So, while Theorem 1 assures that a consonant closed test exists when local Bonferroni tests are used for the testing of each $H\in \mathcal {H}$, not just any graph (*α*,**G**) will result in that consonant closed test. In the following section we demonstrate how a graph (*α*,**G**) can be applied to the GO graph such that a consonant closed test based on weighted Bonferroni tests is achieved through the application of Algorithm 1.

That Algorithm 1, when applied to a graph (*α*,**G**), preserves the monotonic property that *α*_*j*_(*I*)≤*α*_*j*_(*J*) for *I*,*J* such that *H*_*I*_⊂*H*_*J*_ can be seen by noting that Algorithm 1 only provides for the local thresholds *α*_*i*_ to remain the same size or increase. Never does it allow for them to become smaller. Further, at any point in the iterative process, the local thresholds *α*_*i*_ define the weighted Bonferroni test thresholds *α*_*j*_(*I*) for the intersection hypothesis *I* corresponding to the intersection of the elementary hypotheses with non-zero thresholds (see for example Figure 5). Hence, as *H*_*J*_ will be tested only after *H*_*I*_ is first rejected whenever *H*_*I*_⊂*H*_*J*_, it follows that Algorithm 1 will provide *α*_*j*_(*I*)≤*α*_*j*_(*J*).

### The *Short* focus level procedure

We obtain the Short Focus Level procedure by modifying the top-down portion of the Focus Level method. This is done by tailoring the general graphical shortcut [19] to a GO graph as follows. Label the *m* hypotheses corresponding to the test of significance for each GO term (gene set) as *H*_1_,…,*H*_*m*_ starting with the root node and proceeding in an organized manner through each level of the GO graph, ending with the terminal nodes. (The precise ordering is not important.) Let *F*⊂*M*={1,…,*m*} denote the index set of the nodes corresponding to the pre-selected focus level of the GO graph. For all *m*_*F*_ nodes in the focus level, assign local significance levels of *α*_*i*_=*α*/*m*_*F*_ to each hypothesis *H*_*i*_ with *i*∈*F*. Assign initial local significance levels of 0 to all children nodes of the focus level. Note that nodes above the focus level will still be tested using the bottom-up approach of the Focus Level method and are not considered when applying the top-down portion of the method.

Using the structure of the GO graph, assign to each edge from parent node *i* to child node *j* a weight of *g*_*ij*_=1/*m*_*i*_, where *m*_*i*_ denotes the number of children nodes of node *i*. After all edge weights have been assigned for the edges defined by the GO graph, all terminal nodes are individually joined with *m*_*F*_ new edges to each of the *m*_*F*_ focus level nodes. These new edges are given weights of 1/*m*_*F*_. (In the case that a terminal node is also a focus level node, then edges are made only to all other focus level nodes with weight 1/(*m*_*F*_−1)).

**Figure 6:**
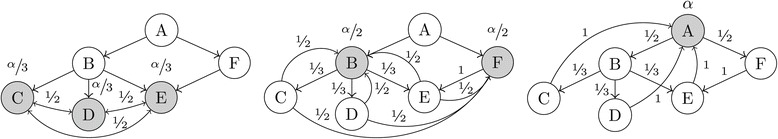
**The suggested shortcut to the top-down portion of the Focus Level method exploits the natural consonance of the weighted Bonferroni tests applied to the GO graph to avoid closing the graph under all unions as in the original top-down approach.**

At this point, a modified form of Algorithm 1 of [19] is applied to the resulting directed graph to obtain the final set of significant hypotheses. The modifications ensure that no child node is tested before all parent nodes are first found significant, maintaining the strong control of the FWER under the restricted hypotheses of the GO graph as well as maintaining Property FL2 of the basic assumptions (or requirements) underlying the Focus Level method (see “Methods” section above). Figure 6 demonstrates the application of the described graphical Bonferroni adjustment to the top-down portion of the Example GO graphs of Figure 1. Comparing Figure 2 to Figure 6 provides a heuristic understanding of how the new top-down approach is computationally faster than the original closure approach because no new nodes need to be created.

An algorithm which implements the Short Focus Level procedure is detailed in Algorithm 2. Here, **H** denotes the index set of testable hypotheses (nodes) and **w**={*w*_*i*_}_*i*∈**H**_ the corresponding set of weights such that *α*/*w*_*i*_ provides the local thresholds *α*_*i*_ for each hypothesis *H*_*i*_ indexed by *i*∈**H**. As described previously, *F*⊂{1,…,*m*} denotes the index set of all pre-selected focus level nodes. The notation **C**_*i*_ denotes the index set of children nodes of the parent hypothesis *H*_*i*_. Similarly, the notations **P**_*i*_ and **A**_*i*_ denote the parents and all ancestors, respectively, of the node corresponding to the hypothesis *H*_*i*_. Finally, we use **R** and **S** to denote the index sets of the current and cumulative rejected hypotheses, respectively.



## Results and discussion

A natural question at this point concerns the advantages and disadvantages of changing the top-down portion of the Focus Level procedure from the original closed test approach as in [10] to the graphical shortcut of [19] as proposed for the Short Focus Level. If the local tests for each intersection hypothesis were originally performed with weighted Bonferroni tests, then the difference between the methods would be that the first performed the full closure test requiring the testing of somewhere on the order of 2^*m*^−1 intersection hypotheses, while the second, which applies a shortcut, would test no more than *m* hypotheses with no reduction in the power of the tests. When using the Global Test for each intersection hypothesis as suggested by [10], the answer to the differences in computation time and power is not as clear. The following simulations demonstrate that neither method is uniformly more powerful than the other, with each having the advantage for certain scenarios. However, as these simulations demonstrate, the newly proposed Short Focus Level procedure is uniformly (and exponentially) computationally faster than the Focus Level method which will hopefully better enable its use by practitioners.

*Simulation 1*

**Figure 7:**
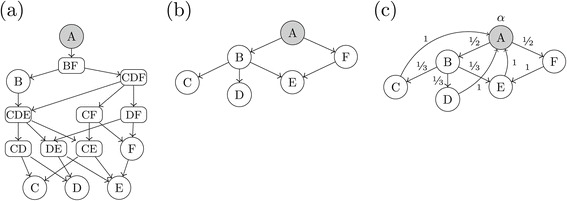
**A toy GO graph example illustrating the difference between the current Focus Level method and the proposed Short Focus Level method.** **(a)** The full closure of the example toy GO graph depicted in panel **(b)** that is currently utilized by the Focus Level method. **(c)** The graph (*α*,**G**) corresponding to the example toy GO graph depicted in panel **(b)** that is utilized by the proposed Short Focus Level procedure.

The following simulation based on the toy GO graph depicted in Figure 7 panel (b) demonstrates the advantages and disadvantages of moving to the newly proposed graphical shortcut of [19] in the top-down portion of the Focus Level procedure. The simulation was performed with the phenotype *Y* as a dichotomous class variable (say, treatment and control) and the data *X* representing an RNA-Seq counts matrix with rows as genes (*m*) and columns as samples (*n*). The number of samples belonging to the treatment group was simulated according to a binomial(*n*, 0.5) distribution, where *n* is the total number of samples, with the added rule that at least two samples were in each group. This allowed for unbalanced data, with the tendency towards fairly balanced designs. Separate simulations for sample sizes of *n*=5, 20, and 100 were performed.

The structure of gene assignments to the sets A, B, C, D, E, and F of Figure 4, as well as the total number of genes assigned, was allowed to vary in each simulation according to certain parameters. Genes were first assigned to the leaf node gene sets C, D, and E. This was accomplished by randomly selecting both the number of distinct sets in each of these sets (anywhere from 1 to a maximum specified size of either 10 or 40) as well as the number of genes shared by all possible combinations of the leaf node gene sets. Common genes between all or many gene sets was discouraged with small probabilities of occurrence, while common genes between a few gene sets was allowed to occur more frequently. Following the assignments of genes to leaf nodes, parent nodes were randomly assigned new genes (anywhere from 1 to the maximum specified size) as well as all genes contained by their children nodes. The result was a nested graph with at least some overlap common to many gene sets, as is the case within GO Graphs.

The data counts matrix *X* was simulated using an actual RNA-Seq data set as a sampling distribution for the per-gene means in the control group. Specifically, the counts *k*_*ij*_ for all samples *j* assigned to the control group were generated from a NB($\mu _{i},\mu _{i}+{\mu _{i}^{2}}/d$) distribution, where the means *μ*_*i*_ were randomly sampled from the per-gene means of the control group from the actual RNA-Seq data set. The scaling parameter *d* was set at 10 for all simulations. Leaf node gene sets (any of nodes C, D, or E in Figure 4) were then selected at random to be significant. Each gene mapping to the selected significant leaf nodes was assigned a treatment mean of $\hat {\mu _{i}} = 2^{\beta _{i}}\mu _{i}$ where *μ*_*i*_ denotes the mean sampled from the actual RNA-Seq data for gene *i* and *β*_*i*_ was an effect size obtained from a Poisson(*λ*) distribution with the parameter *λ* set to one of 0, 1, 2, or 3. Thus, not all genes in the significant gene sets necessarily had non-zero effect sizes. The actual counts *k*_*ij*_ for all samples *j* assigned to the treatment group were obtained from a NB($\hat {\mu }_{i}$, $\hat {\mu }_{i}+\hat {\mu }_{i}^{2}/d$) distribution where, as with the control group, *d*=10 was constant across all simulations. (See [13] for a similar simulation approach where single gene sets were the object of interest as opposed to an entire GO graphs as in this simulation).

The averaged results of Simulation 1 are presented in Table 1. This example shows greater power for the current implementation of the Focus Level procedure where the Globaltest [6] is used to test all intersection hypotheses and all elementary hypotheses. The greatest power differences of the two methods appear for small sample sizes, *n*=5 in this simulation, and for nodes with relatively few child nodes. The power of the two methods is comparable otherwise. Importantly, the computation time for the Short Focus Level procedure is significantly faster, even for this extremely small toy GO graph whose closure contains just 14 nodes. Interestingly, the Focus Level procedure as it is currently implemented seems to operate best, computationally speaking, when the sample size is moderate, *n*=20 in this simulation.

**Table 1:** **Summary of results for Simulation 1**

								**Mean**
		**Node**						**computation**
***n***	**Method**	***A***	***B***	***F***	***C***	***D***	***E***	**time (sec)**
5	FL	0.447	0.428	0.132	0.142	0.135	0.130	0.426134
	SFL	0.447	0.366	0.120	0.092	0.083	0.122	0.001778
20	FL	0.574	0.567	0.180	0.186	0.192	0.179	0.102097
	SFL	0.574	0.552	0.178	0.184	0.188	0.179	0.001789
100	FL	0.642	0.635	0.202	0.220	0.207	0.201	0.355848
	SFL	0.642	0.623	0.201	0.217	0.204	0.201	0.001793

Power calculations were averaged over all levels of the effect size *λ* and both sizes of *m*, the maximum leaf node gene set size, for each level of the sample size *n*.

FL: Focus Level.

SFL: Short Focus Level.

;*Simulation 2;*

**Figure 8:**
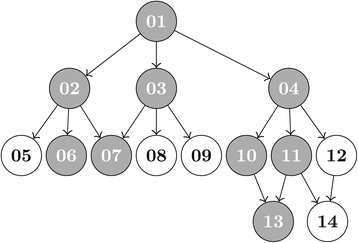
**Structure of the toy GO graph used in** **Simulation 2** **.** Shaded nodes correspond to those GO IDs which were simulated to be significantly associated with the phenotype **Y**.

A second simulation study using the toy GO graph of Figure 8 was also used to compare power and computation time of the original Focus Level method to the Short Focus Level. The closure of the toy GO graph in Figure 8 is more complex than that of the previous simulation, containing 574 nodes as compared to the 14 of Figure 7, panel (a). This simulation considered the continuous phenotype **Y**∼*N*(0,1) and its correlation with simulated gene expression values **X**. For this simulation *m*=100 genes were partitioned to the 14 GO IDs of Figure 8 as specified in Table 2. Expression values *X*_*ij*_ for each sample *i*=1,…,*n* and gene *j*=1,…,*m* were generated as N(0,1) variates. GO IDs 6, 7, and 13 were designated as significant by adding *r***Y**, *r*∈[0,1], to the expression values of the corresponding genes (i.e., the columns of **X** corresponding to genes in GO IDs 6, 7, and 13). Thus, by inherentance, GO IDs 1, 2, 3, 4, 10, and 11 were also significantly associated with the phenotype **Y**. Values of *r* close to 1 provided a strong signal and greater power for detection while *r* near zero resulted in a very weak signal and correspondingly very low power for detection. Goeman’s Globaltest [6] was used to test each GO ID for association with the phenotypic variable **Y**. Given that Simulation 1 suggested that the current Focus Level procedure performs best at a moderate sample size, *n*=20 was used for this simulation.

**Table 2:** **Allocation of simulated genes to the GO IDs of the GO graph in Figure** **8**

GO ID	1	2	3	4	5	6	7
Genes	1-100	1-40	21-60	61-100	1-10	11:20	21:40
GO ID	8	9	10	11	12	13	14
Genes	41-50	51-60	61-80	71-90	81-100	72-79	82-89

Power and computation time were averaged over 1,000 simulations. Results are presented in Table 3 for the most interesting case of *r*=0.5. They show the Short Focus Level method having greater power at every GO ID. The computational speed advantage of the improvement is also manifest, showing nearly a 15,000 fold increase in speed over the current Focus Level procedure. This second simulation emphasizes the fact that neither approach to the Focus Level procedure is uniformly more powerful than the other. While it is clear that each has the advantage in certain scenarios, at least theoretically, more work needs to be completed to determine exactly where each is most appropriate. Practically speaking however, the computational advantage and similar statistical power (on average) of the Short Focus Level should solicit its use except perhaps for choices of the focus level near the leaf nodes of the graph where the current Focus Level method is computationally tractable.

**Table 3:** **Results of the power simulation for the GO graph in Figure** **8**

	**GO:01**	**GO:02**	**GO:03**	**GO:04**	**GO:06**	**GO:07**	**GO:10**	**GO:11**	**GO:13**	**Time**
FL	0.995	0.968	0.890	0.462	0.512	0.872	0.380	0.399	0.344	3:42:938
SFL	0.995	0.988	0.952	0.543	0.837	0.949	0.489	0.476	0.445	0:00:015

FL: Focus Level.

SFL: Short Focus Level.

### Key difference between focus level and short focus level methods

The Focus Level (FL) and Short Focus Level (SFL) methods are the same in the bottom-up approach. They differ in the top-down approach. Both are similar in the top-down approach in that they apply the closed testing strategy to the GO graph (from the focus level down to the leaf nodes) by closing the graph under all unions of gene IDs corresponding to the GO IDs (i.e., intersections of the hypotheses corresponding to the testing of each GO ID). The FL method uses the Global Test to test each intersection hypothesis of the closed GO graph[10]. The SFL method would be a direct shortcut of the FL method if the FL method instead used a weighted Bonferroni test to test each intersection hypothesis.

However, it is important to recognize that the FL method could only perform the full closure test, not the short-cut that the SFL method performs, even if the FL method was modified to use the weighted Bonferroni tests. The FL method is more consistent in applying the same Global Test to both original GO ID hypotheses as well as to all intersection hypotheses. However, it is computationally expensive because it performs the full closure test. The SFL method makes a slight shift in allowing any test (not just the Global Test) for the elementary hypotheses (the individual GO ID hypotheses) and then performing weighted Bonferroni tests for all intersection hypotheses. This simplification or generalization allows for the resulting short-cut. Hence, the power comparison between the FL and SFL methods is not obvious, and the simulations above show that neither method is uniformly more powerful than the other.

### Real data application 1

A drawback to the otherwise powerful Focus Level method is the computational burden which prohibits the full top-down approach from being applied to real data sets [10]. When no *a priori* focus level exists, as is often the case [18], the root node of the GO graph is a logical default choice, but requires the full top-down approach. Under the newly proposed Short Focus Level method, this is now a computational possibility. The following application to RNA-Seq counts data from porcine oocytes demonstrates the performance of the full top-down approach of the Short Focus Level procedure to real data. The Biological Process (BP) root node was selected as the focus level for this study due to there being no other focus level of greater *a priori* interest.

It is well known that *in vivo* (naturally) maturated oocytes show far greater developmental competence than do those matured *in vitro* [31]. Yet, the underlying genetics are still not well understood. To uncover the genetic differences of *in vitro* matured oocytes as compared to those matured naturally (*in vivo*), transcript counts for 4 *in vivo* and 4 *in vitro* maturated porcine oocytes were obtained using the Illumina RNA-Seq platform [32]. Lanes were populated as shown in Table 4. These data from the lab of Dr. Clay Isom of the Utah State University Department of Animal, Dairy, and Veterinary Sciences are reported on here with permission. In this oocyte study, all animal procedures were performed with the strictest adherence to animal welfare guidelines and with regulatory oversight by the Institutional Animal Care and Use Committee at Utah State University.

**Table 4:** **Experimental design for the** ***in vivo*** **(IVV) and** ***in vitro*** **(IVM) oocyte maturation RNA-seq data**

**Oocyte no.**	**Embryo type**	**Pig (Mother)**
1	IVV	1
2	IVV	2
3*	IVV	3
4	IVM	3
5	IVM	1
6	IVM	2
7	IVM	4
8	IVV	3

^*^Lane 3 contained quality problems and was removed from the analysis.

Individual *P*-values testing the differential expression of 12,625 genes were calculated using the DESeq package of Bioconductor [33,34] with pig mother, as identified in Table 4, included as a covariate. Specifically, these *P*-values were obtained under the null hypotheses that the per-gene expression strength of the *in vivo* maturated oocytes (IVV) is equal to that of the *in vitro* maturated oocytes (IVV) when accounting for any pig mother effect. This was done through the DESeq package [33] which compares a full model (regressing the RNA-Seq counts on both the oocyte type and pig mother by a generalized linear model) to a reduced model (regressing only on the pig mother) to determine significance for a given gene.

A gene set analysis using the GO BP ontology was then performed to characterize differentially expressed gene products between the two types of oocytes (IVV and IVM). *P*-values for each of 5,687 BP GO Terms containing at least 5 of the 12,625 Entrez IDs from the single gene (DESeq) analysis were calculated using Stouffer’s Method [24,35]. The R code [21] for Stouffer’s Method is included in the mvGST package [22,23]; see also Additional file 1. Briefly, Stouffer’s method transforms each of the *P*-values (from the single gene analysis) corresponding to an individual gene in the gene set to a standard normal *Z*-score. A single *P*-value for the gene set is then obtained from the mean of the *Z*-scores by computing the appropriate tail probability (from a standard normal distribution) beyond the mean *Z*-score. Stouffer’s *P*-value combination method was applied here as it is more powerful for the consensus alternative than say Fisher’s *P*-value combination test [36] or Goeman’s globaltest [6], see discussions in [24]. Finally, multiplicity adjusted gene set *P*-values for each BP term were calculated using the Short Focus Level procedure, with the root BP GO term (GO:0008510) as the focus level. This adjustment (the full top-down approach) took just 3 minutes and 23 seconds of processing time on an Intel Pentium M 1.86 GHz processor with 1 GB of RAM. The current Focus Level method is computationally intractable for thesedata.

**Figure 9:**
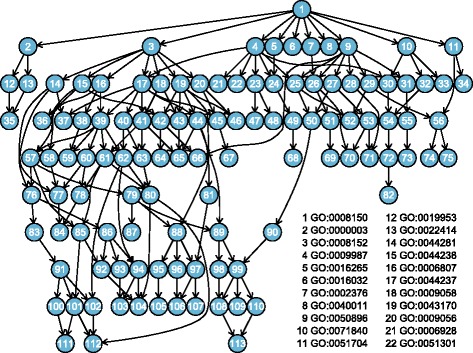
**Significant results from the gene set testing of porcine oocytes obtained from the Short Focus Level procedure using the full top down approach.**

Figure 9 reports the significant subgraph [10] obtained from the Short Focus Level method containing 113 of the original 5,687 BP terms. While a partial legend is included in Figure 9, a full legend identifying all 113 significant BP terms, along with their multiplicity-adjusted *P*-values (using the Short Focus Level method at familywise error rate 0.05) is included as a table in Additional file 3. Since the full top-down approach was performed, these GO terms, which are differentially expressed between the two types of oocytes (IVV and IVM), can be discussed either individually or within their context of this significant subgraph. Advantaged by the FWER control of the Short Focus Level procedure, any subset of the significant results can also be reported on (while the others ignored) with the assurance that the FWER remains controlled at the specified level for the selected sets. Possible interpretation discussions of the results include the significant differential activity (between *in vivo* and *in vitro* maturated oocytes) of biological processes “response to bacterium” (node 74 in Figure 9), “double-strand break repair” (node 110), and “ribonucleoside metabolic process” (node 93), among others.

### Real data application 2

As a second real data demonstration of the Short Focus Level method, and to further discuss differences between the Short Focus Level and Focus Level methods, we used a subset of the famous Golub data set [37], specifically the 38 training samples publicly available in the R package golubEsets [38]. Briefly, Affymetrix Hgu6800 chips were used to profile gene expression in leukemia patients, and we test for differential biological process activity between 27 patients with acute lymphoblastic leukemia (ALL) and 11 with acute myeloid leukemia (AML). Using the same Focus Level method demonstration as in the vignette of the globaltest package [39], we looked only at biological process “cell cycle” (GO:0007049) and its descendants in the GO graph, with 249 total nodes.

We also applied the Short Focus Level method to the same example (using familywise error rate 0.01), and note that while the Focus Level method (with its default focus level) takes nearly 4 minutes, the Short Focus Level method (using the same focus level as selected by the Focus Level method) takes 1 second. Finally, we applied the Short Focus Level method using the root node as the focus level, which also took 1 second. In stark contrast, the Focus Level method using the root node as the focus level was deemed computationally intractable, as even a run time of two weeks was not sufficient to complete it.

**Figure 10:**
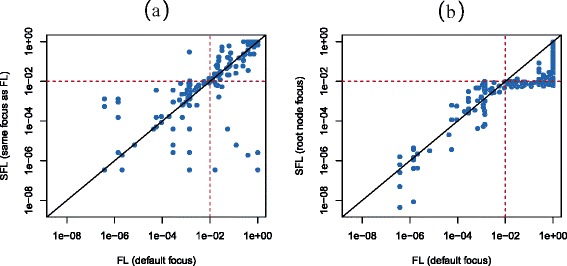
**Adjusted p-values for each of the 249 biological processes considered in the Golub example.** The Focus Level (FL) method with its default focus level is compared to the Short Focus Level (SFL) method using **(a)** the same focus level as the FL method and **(b)** the root node focus level (which is computationally intractable for the FL method). Red dashed lines correspond to the familywise error rate of 0.01, and the solid black line represents the line of equality. All axes are on the log scale.

Figure 10 compares the resulting adjusted p-values for each of the 249 biological processes considered. Figure 10a shows that, when using the same focus level, the Focus Level (FL) and Short Focus Level (SFL) methods can result in different (though largely overlapping in this case) sets of GO terms called significant. This results from the previously discussed key difference between the FL and SFL methods, namely that the SFL method allows any test (not just the Global Test) for the elementary hypotheses (the individual GO ID hypotheses) and then performs weighted Bonferroni tests for all intersection hypotheses.

As discussed in the original Focus Level paper [10], different focus levels provide for different power at differing areas of the GO graph. For this reason, it is difficult to make definitive comparisons using results from different focus levels. While the stronger agreement seen between the FL method (with its default focus level) and SFL method (with the root node focus level) in Figure 10 may seem interesting, the important point is that the FL method is effectively computationally intractable using the root node focus level. The decision to use the FL or SFL method should not be based on power considerations, but rather on computational considerations, especially when no real reason exists to choose the focus level anywhere other than the root node (which will most often be the case), in which case the FL method is computationally intractable. However, the SFL method is computationally efficient and strongly controls the familywise error rate within the structure of the GO graph.

## Conclusions

As pointed out in [10], the GO graphs are structured and “it is wasteful not to make use of that structure” in correcting for multiplicity. Further, they stress the importance of not making any assumptions on the joint distribution of the test statistics corresponding to each of the gene sets in the GO graph while correcting for multiplicity. The Focus Level procedure both avoids any such assumptions and capitalizes on the inherent structure of the GO graph to adjust for the multiple tests performed, resulting in a powerful approach. Another advantage of the Focus Level method is the possibility of incorporating biological knowledge into the adjustment approach through the selection of the focus level, where the method has the greatest power.

This work improves upon the Focus Level procedure of [10] by altering the top-down portion of the method to utilize the graphical shortcut of [19] in place of the full closed testing approach of [17] as originally suggested by [10]. This was made possible by extending the result from [20] to restricted hypotheses (Theorem 1) as the hypotheses corresponding to the GO graph are always restricted.

The main advantage of the Short Focus Level procedure proposed in this work is the exponential decrease in computational burden. This provides for the most logical default choice of the root node of the GO graph as the focus level when no other *a priori* choice can be specified. Another advantage of the improvement is in the ability to consider the adjusted *P*-values apart from their context within the significant subgraph of the full GO graph under the full top-down approach. When the focus level is selected to be anything other than the root node, individual hypotheses must be considered in context of their position within the significant subgraph. However, this is not altogether a disadvantage as “the interpretation of an individual adjusted P-value should depend on the location in the graph where it occurs” [10].

It is our hope that this shortcut for the Focus Level procedure, the Short Focus Level, will result in more wide-spread use of the method. Still, future work remains to be done. The simulations performed within this work demonstrate that each approach appears to be more powerful under different circumstances. Hence, further theoretical work is needed to determine the conditions under which each method is most powerful.

## Additional files

Additional file 1
**Short focus level and Stouffer’s P-value combination R code.**

Additional file 2
**Short focus level R code help file.**

Additional file 3
**Full legend to Figure** 9.
