# Supplementary material for: A shortcut for multiple testing on the directed acyclic graph of gene ontology
Source: BMC Bioinformatics. 2014 Nov 1;15(1):349. doi: 10.1186/s12859-014-0349-3 (PMC4232707; doi:10.1186/s12859-014-0349-3)
Supplement: Additional file 2 — Short focus level R code help file. [file 12859_2014_349_MOESM2_ESM.zip › 6458614211299644_add2.htm]

R: Short Focus Level adjustment

|  |  |
| --- | --- |
| p.adjust.SFL {mvGST} | R Documentation |

## Short Focus Level adjustment

### Description

Takes a named numeric vector of raw p-values as input
and returns the Short Focus Level adjusted p-values,
where the adjustment is based on controlling the FWER
at a specified level within the structure of the GO graph.

### Usage

```
p.adjust.SFL(rawp, ontology=c("BP","CC","MF"), 
    focus='rn', ancestors, offspring, trace=FALSE, 
    recycle=TRUE, sig.level=0.05)
```

### Arguments

|  |  |
| --- | --- |
| `rawp` | named numeric vector of p-values where the names correspond to the GO ID for which the provided p-values correspond to the given GO Term. These must be 'two-sided' p-values, i.e., from a two-sided test. |
| `ontology` | The ontology of interest. Must be one of 'BP', 'CC', or 'MF'. All names of rawp must be from the same ontology. Defaults to 'BP'. |
| `focus` | the focus level of interest. Default is set at the root node, the logical place to start if there is no better place to start. |
| `ancestors` | named lists corresponding to the ancestor and offspring structure of all named GO IDs in rawp. These are optional, and simply result in faster computation of the adjusted p-values if they are already available from the R session when p.adjust.SFL is called. |
| `offspring` | named lists corresponding to the ancestor and offspring structure of all named GO IDs in rawp. These are optional, and simply result in faster computation of the adjusted p-values if they are already available from the R session when p.adjust.SFL is called. |
| `trace` | logical denoting whether or not progress about the algorithm is output to the user. Defaults to FALSE. |
| `recycle` | logical determining whether or not to recycle any threshold corresponding to a rejected leaf node back into the GO graph. Defaults to TRUE, as it can result in greater power for the Short Focus Level method at a slight increase to the computational burden. |
| `sig.level` | numeric value at which to control the family-wise error rate within the structure of the GO graph. |

### Value

returns the adjusted p-values with naming and ordering
identical to the original "rawp" values.

### Author(s)

John R. Stevens and Garrett Saunders

### References

Saunders G., Stevens J.R., and Isom S.C. "A shortcut for multiple testing
on the directed acyclic graph of Gene Ontology." BMC Bioinformatics 2014
(under review).

Saunders, G., 2014. "Family-wise error rate control in QTL mapping and
gene ontology graphs with remarks on family selection." PhD thesis,
Utah State University, Department of Mathematics and Statistics.
http://digitalcommons.usu.edu/etd/2164/

### Examples

```
# Get GO terms of interest
library(GOstats); library(annotate)
GO.vec <- c("GO:0001775","GO:0007275")
g <- GOGraph(GO.vec, GOBPPARENTS)
g <- removeNode("all",g)
GOids <- names(nodes(g))

# Get p-values for all GO terms of interest
# (here, simulated for demonstration)
# Make sure names are GO term IDs
set.seed(1)
rawp <- rbeta(length(GOids), .2, 1)
names(rawp) <- GOids

# P-value adjustment using Short Focus Level
# -- This p.adjust.SFL function will be included
#    in forthcoming mvGST package. In the meantime,
#    see Additional Files of Saunders, Stevens, and Isom (2014).
padj <- p.adjust.SFL(rawp, ontology='BP')
head(padj)
# These are in the same order as rawp, with
# names corresponding to GO terms.
# Calling GO terms significant when padj is 
# less than alpha controls the FWER at alpha,
# within the context of the GO graph.
```

---

[Package *mvGST* version 0.99.2 ]
